# Immunization with one *Theileria parva* strain results in similar level of CTL strain-specificity and protection compared to immunization with the three-component Muguga cocktail in MHC-matched animals

**DOI:** 10.1186/s12917-018-1460-x

**Published:** 2018-05-02

**Authors:** Lucilla Steinaa, Nicholas Svitek, Elias Awino, Thomas Njoroge, Rosemary Saya, Ivan Morrison, Philip Toye

**Affiliations:** 1 International Livestock Research Institute, P.O. Box 30709, Nairobi, 00100 Kenya; 2 The Roslin Institute, The University of Edinburgh, Midlothian, EH25 9RG UK

**Keywords:** *Theileria parva*, Live vaccine, Cytotoxic T cells, Immunity, Strain specificity

## Abstract

**Background:**

The tick-borne protozoan parasite *Theileria parva* causes a usually fatal cattle disease known as East Coast fever in sub-Saharan Africa, with devastating consequences for poor small-holder farmers. Immunity to *T. parva,* believed to be mediated by a cytotoxic T lymphocyte (CTL) response, is induced following natural infection and after vaccination with a live vaccine, known as the Infection and Treatment Method (ITM). The most commonly used version of ITM is a combination of parasites derived from three isolates (Muguga, Kiambu 5 and Serengeti-transformed), known as the “Muguga cocktail”. The use of a vaccine comprising several strains is believed to be required to induce a broad immune response effective against field challenge. In this study we investigated whether immunization with the Muguga cocktail induces a broader CTL response than immunization with a single strain (Muguga).

**Results:**

Four MHC haplotype-matched pairs of cattle were immunized with either the trivalent Muguga cocktail or the single Muguga strain. CTL specificity was assessed on a panel of five different strains, and clonal responses to these strains were also assessed in one of the MHC-matched pairs. We did not find evidence for a broader CTL response in animals immunized with the Muguga cocktail compared to those immunized with the Muguga strain alone, in either the bulk or clonal CTL analyses. This was supported by an in vivo trial in which all vaccinated animals survived challenge with a lethal dose of the Muguga cocktail vaccine stabilate.

**Conclusion:**

We did not observe any substantial differences in the immunity generated from animals immunized with either Muguga alone or the Muguga cocktail in the animals tested here, corroborating earlier results showing limited antigenic diversity in the Muguga cocktail. These results may warrant further field studies using single *T. parva* strains as future vaccine candidates.

## Background

*Theileria parva* is a tick-borne protozoan parasite which causes an acute and usually fatal cattle disease, known as East Coast fever, in eastern, central and southern Africa. The parasite infects bovine lymphocytes, which subsequently undergo blast transformation and rapid multiplication [1]. In susceptible animals, this usually results in overwhelming parasitosis and death within 2 to 4 weeks of infection.

Cattle which recover from natural infection can develop a strong immunity to subsequent challenge. This has been exploited to develop a vaccination procedure known as the “Infection and Treatment Method” (ITM) in which live sporozoites are administered simultaneously with oxytetracycline. The main protective mechanism in both vaccinated and naturally recovered animals is believed to be CD8+ cytotoxic T lymphocyte (CTL) killing of infected lymphocytes. Thus, adoptive transfer of CD8^+^ cells from immunized animals has been demonstrated to protect naïve animals from challenge [2]. Furthermore, it has been shown that the time point of recovery correlates with a peak of CD8^+^ cells in the blood of the infected animals [3].

Early experiments in the development of the ITM vaccine revealed the presence of strain specificity through infection and challenge trials where animals were immunized with one strain and challenged with a heterologous strain. While good protection was often obtained following the inoculation of single strains, it did not always extend to heterologous challenge [4]. It was subsequently shown that a combination of three strains (Muguga, Serengeti-transformed and Kiambu 5) provided better protection than single strains. The mixture, known as the “Muguga cocktail”, is the basis of a commercial ITM vaccine which appears to provide broad protection against *T. parva* in the field, so far best explored in Tanzania [5, 6]. It should be noted that in experiments involving single isolates, some cross-protection was observed with a notable exception being if animals vaccinated with the Muguga strain or Kilifi strain were challenged with Marikebuni [4, 7, 8], suggesting that Marikebuni is antigenically quite distinct.

Strain specificity has also been observed in in vitro CTL assays and reflects the in vivo immune status [3, 9–11]. A possible explanation for the strain specificity of the CTL response to *T. parva* is based on two phenomena – immunodominance and antigenic diversity. Immunodominance, where the immune response is directed to a very limited number of antigens in individual animals, is commonly observed in viral infections [12–15] and also in other infectious diseases and cancers [16–18]. In *T. parva* infections, indications of immunodominance came with initial analyses of the immune response where, in some cases, it was possible to show that CTL restriction was mediated by a single class I MHC molecule [19, 20] despite the presence of many potential epitopes expressed by the parasite genome, which is predicted to encode 4034 genes [21, 22], and a T cell receptor (TCR) repertoire capable of reacting to a wide variety of epitopes. More recently, with the discovery of CTL antigens [23], a study was performed to shed light on the issue of immunodominance [24]. Findings from this work suggest that one or a few MHC alleles in individual animals govern the specificity of the immune response elicited by infection or vaccination and are crucial for the outcome of a later challenge with genotypically different parasites.

The most plausible explanation for the broad protection offered by the Muguga cocktail, is that the mixture of the parasites in the cocktail, provides a more diverse set of antigens which potentially can induce a CTL response of broader antigenic specificity than inoculation with any of the individual components or single strains, and thus provide better protection against heterologous parasites encountered in the field. To test this hypothesis, we compared the specificities for different parasite strains of CTL induced in animals immunized by ITM with the Muguga cocktail with those generated by the Muguga stabilate alone. To minimize any effect of the MHC background in individual animals, which is known to influence the selection of antigens recognized by the CTLs, the responses were compared in MHC haploidentical pairs of cattle. Analysis of the CTL response at the clonal level was also undertaken for one haplotype matched pair. All haploidentical pairs of cattle were challenged with the Muguga cocktail to uncover any antigenic differences in the in vivo CTL response.

## Methods

### Animals and MHC class I typing

All animal experiments were reviewed and approved by the Institutional Animal Care and Use Committee at International Livestock Research Institute (ILRI). Eight *Bos taurus* cattle (five Friesian and three Ayrshire) were bought from farms in the Nyeri area in Kenya. They were screened free for tickborne diseases including *T. parva,* and BoLA typed using a combination of serology (ELISA using antibodies defining particular MHC haplotypes), IFNγ ELISPOT assay using PBMC from the cattle pulsed with Tp1_214–224_ and a peptide-specific CTL line, and by PCR using haplotype specific primers followed by sequencing, essentially as described before [25]. Four haploidentical pairs of the following MHC haplotypes were selected for the study: A10/A12, A12/A14, A15/ A18, A11/A15 (Table 1). Animals were kept in standard pens and were fed normally. At the end of the experiment, animals were returned to the farm and eventually slaughtered for meat. Two control animals, which developed disease, were euthanized for humane reasons using an overdose of Euthatal (Pentobarbital sodium, 200 mg/ml), 1 ml Euthatal per 1.4 kg body weight, given intravenously, after restraining the animals.

**Table 1:** Cattle used in the study

Calf	Breed	MHC Class 1 Haplotype	Alleles	Immunization
BG033	Friesian	A10/A12	N*00201, N*01901	Muguga (3308)
BG042	Friesian	A10/A12	N*00201, N*01901	Muguga cocktail (0801)
BG053	Ayrshire	A12/A14	N*01901, N*02301	Muguga (3308)
BG051	Ayrshire	A12/A14	N*01901, N*02301	Muguga cocktail (0801)
BG052	Friesian	A15/A18	N*00901, N*01302	Muguga (3308)
BG056	Friesian	A15/A18	N*00901, N*01302	Muguga cocktail (0801)
BH055	Ayrshire	A11/A15	N*01802, N*00902	Muguga (3308)
BH047	Friesian	A11/A15	N*01802, N*00902	Muguga cocktail (0801)

The breed, MHC Class I haplotype and associated MHC alleles of the cattle included in the study are shown. Within each of the haplotype matched pairs of cattle, one was immunized with the single *T. parva* strain Muguga 3308 and the other was immunized with the Muguga cocktail 0801 using ITM

### Immunization

Each of the haplotype-matched pairs of cattle was inoculated subcutaneously in front and below the right ear with either the Muguga stabilate 3308 or the Muguga cocktail vaccine stabilate ILRI0801 [26] and treated immediately with long acting oxytetracyclin.

### Parasitized cell lines

Cell lines infected with *T. parva* were established by infection of autologous PBMC in vitro with sporozoites as described previously [27]. The sporozoites were from the cloned stabilates Marikebuni 3292, Muguga 3308, Boleni 3230, Uganda 3645, derived from 3569 [28], and Mariakani 3212 (unpublished). In addition, cell lines were established using sporozoites of the ILRI0801 reference stabilates: Muguga (4230), Serengeti (4229), Kiambu 5 (4228). These are stabilates of the individual Muguga cocktail components made from the same production ticks as the ILRI0801 vaccine [26].

### Generation of CTL

CTL bulk cultures were generated and maintained in RPMI 1640 (Sigma-Aldrich, St.Louis, MO, USA) supplemented with 10% FBS (Thermo Fisher Scientific, Waltham, MA, USA), 2 mM L-glutamin (Sigma-Aldrich, St.Louis, MO, USA), 50 μM 2-mercaptoethanol (Sigma-Aldrich, St.Louis, MO, USA), 100 IU of penicillin/ml (Sigma-Aldrich, St.Louis, MO, USA), 100 μg of streptomycin/ml (Sigma-Aldrich, St.Louis, MO, USA), 50 μg of gentamicin/ml, (Sigma-Aldrich, St.Louis, MO, USA) 10% TCGF (Conditioned media from ConA blasts).

CTL were generated essentially as described [27, 29]. Briefly, PBMC were re-stimulated three times with irradiated autologous *T. parva*-infected cell lines. CTL from animals immunized with the vaccine were generated by stimulating with equal fractions of three cell lines infected with one of the three vaccine reference stabilates. CTL from the Muguga- immunized animals were stimulated with the Muguga-infected cell line only. The remainder of the procedure was as described previously [27, 29].

### Generation of CD8^+^ CTL clones

Clones were generated by purifying CD8^+^ cells after two restimulations as described above. Enrichment of CD8^+^ cells was achieved by incubating the CTL line for 30 min with mouse-anti-bovine CD8 mAb ILA105 (ILRI) diluted 1:500 in PBS + 2% FBS. Cells were washed twice in PBS + 2%FBS, labeled with magnetic beads attached to goat-anti-mIgG (Miltenyi Biotec, Bergish Gladbach, Germany) and purified according to the protocol provided by the manufacturer. Purified CD8^+^ cells were then seeded by limiting dilutions in 96-well plates using 2 × 10^4^ irradiated PBMC as filler cells as previously described [27].

CTL lines and clones were generated and maintained in RPMI 1640 medium (Sigma-Aldrich, St. Louis, MO, USA) supplemented with 10% FBS (Thermo Fisher Scientific, Waltham, MA, USA), 2 mM L-glutamine, 50 μM 2-mercaptoethanol, 100 IU/ml of penicillin, 100 μg/ml of streptomycin, 50 μg/ml of gentamicin, (all from Sigma-Aldrich, St. Louis, MO, USA) and 10% inactivated ConA supernatant (conditioned media from ConA blasts).

### Cytotoxicity assay

A standard 4 h release assay using ^51^Cr-labeled target cells was used to measure cytotoxicity. ^51^Cr was obtained from American Radiolabeled Chemicals, Inc., St. Louis, MO, USA. Supernatants were counted using Lumaplates (PerkinElmer, Waltham, MA, USA) in a TopCounter (PerkinElmer, Waltham, MA, USA). The cytotoxicity was calculated as: (experimental release-spontaneous release/total release-spontaneous release). Target cells were either *T. parva*-infected autologous PBMC using MHC-mismatched *T. parva*-infected cell lines as controls. In one case (BH055), autologous uninfected PBMC were used as control. Each CTL was tested in dilutions using a fixed number of target cells. Each dilution was tested in triplicate. Cytotoxicity at an effector:target ratio of 10:1 was used to compare between CTL derived from different animals and between different target cells.

### Challenge experiment

Cattle were challenged with the Muguga cocktail vaccine stabilate ILRI0801, without oxytetracycline. The vaccine (2 ml neat) was injected subcutaneously in front of and below the right ear. Animals were monitored for the required clinical parameters to determine the severity of disease according to the Rowlands index [30].

## Results

### Similar strain specificities of CTL from haplotype-matched animals immunized by ITM with either *T. parva* Muguga or the Muguga cocktail

Eight cattle comprising four haploidentical pairs were immunized by ITM with either the Muguga strain (3308) or the trivalent Muguga cocktail (ILRI0801), as detailed in Table 1.

**Fig. 1:**
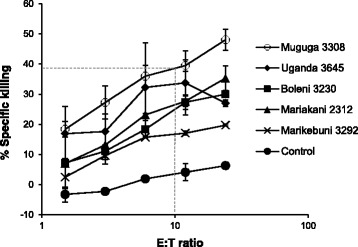
Example of the result from a CTL assay. Serial dilutions of CTL from BG052 were tested for lysis of fixed numbers of the various target cells shown in the figure. The dashed line represents the deduced specific killing at an effector:target ratio (E:T ratio) of 10:1 for the target Muguga 3308. This method was used to compare killing of the various target cells as listed in Table 2. Each point represents the average of a double-determination with the SD shown

CTL were generated from all animals and tested against autologous cell lines infected with five cloned sporozoite stabilates and an MHC-mismatched infected cell line as control. Figure 1 shows an example of the results for a CTL assay of one of the animals. For simplicity, the cytotoxicity for all animals at the same effector/target ratio of 10:1 was deduced from dilution curves, as shown in Fig. 1. The results for all animals, as shown in Table 2, allows a comparison of the breadth of specificity to five different parasite strains exhibited by CTL from the Muguga-immunized and the Muguga cocktail-immunized calves. ANOVA analysis confirmed that there were no statistically difference (*P* = 0.421). Importantly, CTL from the Muguga-immunized animals killed all five targets to various degrees and no clear strain specificity was observed. CTL from BH047 showed a consistently lower level of killing compared to the other CTL, despite several attempts to establish a bulk culture showing higher cytotoxicity.

**Table 2:** Cytotoxic T cell responses in immunized cattle assayed on target cells infected with different cloned *T. parva* strains

Muguga	Uganda 3308	Mariakani 3645	Boleni 3212	Marikebuni 3230	Control 3292	
BG033 (M)	34	13	35	16	ND	0
BG042 (C)	26	18	17	9	18	0
BG053 (M)	59	40	14	20	34	0
BG051 (C)	51	25	38	22	28	3
BG052 (M)	38	33	25	23	17	4
BG056 (C)	3	22	20	11	13	3
BH055 (M)	40	30	26	13	20	0^a^
BH047 (C)	10	8	8	7	4	0

Cytotoxic T cell responses using autologous PBMC infected with different cloned *T. parva* strains as target cells. The specific cytotoxicity at effector/target ratios of 10:1 are shown. Each value is deduced from an effector (CTL) titration curve as shown in Fig. 1. (M) Muguga 3308 immunized; (C) Muguga cocktail immunized. (ND) Not determined, (^a^) Control was autologous PBMC. Relative differences of the CTL specificities, between M and C, were tested using ANOVA analysis (*P* = 0.421)

CTL from the Muguga cocktail-immunized animals were also assessed for recognition of all three components present in the Muguga cocktail. As seen in Table 3, all Muguga cocktail-immunized animals showed killing of the three components. Interestingly, the Muguga component was killed more effectively in all cases (*P* < 0.001 for each of the cattle) except BH047, where the killing of the Serengeti component was slightly greater (not statistically significant). As there can be variability in CTL assays, these experiments were done at least twice, and for some animals three times, and the results were similar.

**Table 3:** Cytotoxic T cell responses in cattle immunized with the Muguga cocktail on the three *T. parva* component strains as targets

	Muguga 4230	Serengeti 4229	Kiambu 5 4228	Control
BG042	26^a^	18	13	0
BG051	40^a^	28	34	0
BG056	27^a^	22	15	0
BH047	9	11	5	0

Cytotoxic T cell responses by vaccine immunized animals using autologous PBMC infected with the three different *T. parva* component strains as targets. Each CTL was titrated on a fixed number of target cells. The effector/target ratio of 10:1 is shown and each value is deduced from an effector (CTL) titration curve as shown in Fig. 1. Statistical comparison of parameters from fitted curves were used to test differences in cytotoxicity between targets. (^a^) significant higher than for both other targets for each animal (*p* < 0.001)

### Cloned CTL from a haplotype-matched pair show similar strain specificities

**Fig. 2:**
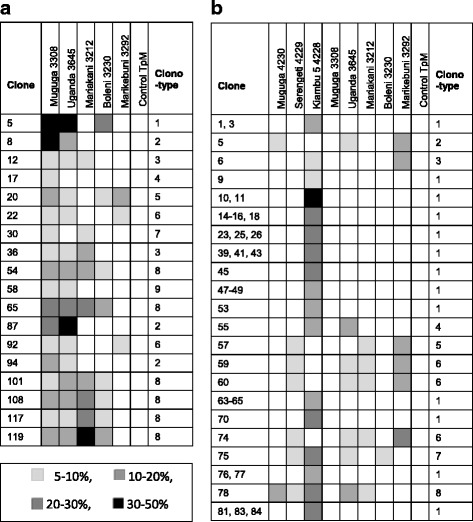
Cytotoxicity obtained by T cell clones on a panel of target cells infected with different cloned *T. parva* strains. The cutoff value was 5% cytotoxicity. Clones were categorized into clonotypes based on their pattern of reactivity. The level of cytotoxicity is visualized as a heat map – colour codes are shown. **a** Calf BG053 immunized with Muguga 3308. CD8 T cell clones were generated from CD8-purified bulk cultures and tested for cytotoxicity to 5 different strains (as shown) and a MHC-mismatched control TpM. **b** The haplotype-matched calf BG051 immunized with the Muguga cocktail 0801. Clones were tested for cytotoxicity on the same target cells as BG053 and the additional components of the Muguga Cocktail

To examine the CTL specificity in more detail, we generated CTL clones from one haploidentical pair, namely the A12/A14 pair of BG053 and BG051. We also successfully generated clones from BG052 (results not shown) but not from the rest of the animals. Each clone was tested for cytotoxicity using the five different cloned sporozoite strains as target cells. Figure 2a shows a heat map of the clonal analysis of BG053 which was immunized with Muguga 3308. It is clear from the pattern of recognition that nine different clonotypes from BG053 were identified, with some clones recognizing only one or two strains, and others recognizing three or four of the five strains. The least recognized strain was Marikebuni, and only one clone was found that recognized Muguga alone. In general, the clonal analysis corresponded well with the bulk analysis, where strongest killing was also observed with the Muguga and Uganda stabilates. On the other hand, few of the clones recognized Marikebuni in contrast to the results observed with the bulk cell lines.

The clonal analysis of the Muguga cocktail-immunized animal BG051 is shown in Fig. 2b. Eight clonotypes were identified, with a surprisingly high number of clones specific for Kiambu5 only. There were also clones which recognized a broader set of targets. As observed in the analysis of bulk CTL lines, there was no evidence from the clonal analysis of a wider set of clonal reactivities in the Muguga cocktail-immunized animal compared to the Muguga only-immunized animal. Table 4 shows the number of clones with the percentages in brackets, that recognize indicated numbers of different cloned strains (upper part), and the number of clones with percentage in brackets recognizing a particular parasite strain (lower part). It is evident that a large fraction of clones from the Muguga cocktail-immunized animal recognized one strain only, whereas more clones from the Muguga only-immunized animal recognized many of the strains, which is opposite of what was expected. All clones from the Muguga only-immunized animal recognized Muguga as expected, and 89% recognized Uganda, implying that this strain is quite similar to Muguga. On the other hand, Marikebuni was the least recognized strain, being specifically lysed by only 17% of the clones. In the Muguga cocktail-immunized animal, 87% of the clones recognized Kiambu5, one of the components in the vaccine, compared to 5% and 16% that recognized the Muguga and Serengeti-transformed components, respectively. Interestingly, there was a difference in the two Muguga-infected targets, which may reflect qualitative differences in presented epitopes in these two target cells.

**Table 4:** Number and percentages of CTL clones from BH053 and BH051 recognizing multiple *T. parva* strains

Calf	BG053 (M)	BG051 (C)
Total	18	38
1 strain	1 (6)	29 (76)
2 strains	5 (28)	2 (5)
3 strains	5 (28)	2 (5)
4 strains	7 (39)	4 (11)
5 strains	0 (0)	1 (3)
Muguga (4230)	ND	2 (5)
Serengeti (4229)	ND	6 (16)
Kiambu-5 (4228)	ND	33 (87)
Muguga (3308)	18 (100)	2 (5)
Uganda (3645)	16 (89)	7 (18)
Mariakani (3212)	9 (50)	5 (13)
Boleni (3230)	8 (44)	1 (3)
Marikebuni (3292)	3 (17)	6 (16)

Upper part of the table: The total number of clones with percentages in brackets, that recognize multiple cloned *T. parva* strains, with a cutoff value of 5% cytotoxicity, for the haplotype matched pair, BG053 (Muguga immunized) and BG051 (Muguga cocktail immunized), is shown. Each clone was analyzed for the number of strains that it recognized. The left numbers in each column represents the actual number of clones recognizing 1 strain, 2 strains, etc. The numbers in brackets represents the corresponding percentages of clones recognizing 1 strain, 2 strains etc. Percentages have been rounded. Lower part of the table: The total number of clones and percentages in brackets from BG053 and BG051 recognizing the different *T. parva* strains. Percentages have been rounded. BH051 was tested on the Muguga cocktail component reference stabilate strains in addition to the cloned strains. (ND) Not determined

In summary, the results of the CTL assays do not support the hypothesis that immunization with the Muguga cocktail induces CTL with a broader reactivity against *T. parva*-infected cell lines compared to immunization with the Muguga stabilate alone, at least with the parasite strains and MHC haplotypes of the animals assessed here.

### Similar protection to the vaccine strains in animals vaccinated with Muguga or the Muguga cocktail

The animals were challenged with the Muguga cocktail in order to investigate if there were any differences between the Muguga-immunized animals and the Muguga cocktail-immunized animals in their immunity to parasite strains present in the Muguga cocktail vaccine but not in the Muguga stabilate. Two non-immunized control animals were used to confirm a sufficient challenge had been delivered and the clinical outcome was assessed with the Rowlands ECF index [30]. The experiment was stopped at day 14 as there was no development of disease in the animals except for the two control animals. As seen in Table 5, there was no substantive difference in the protection to the Muguga cocktail between animals immunized with Muguga only or the Muguga cocktail. Only one animal from each immunized group developed pyrexia, and schizonts were detected in three of the seven animals, two from the Muguga-immunized group and one from the Muguga cocktail-immunized group. This contrasts strongly with the control animals, both of which developed pyrexia and had detectable schizonts and piroplasms. Interestingly, the animal with the highest ECF score, BH047, was the animal with the weakest CTL response.

**Table 5:** Challenge of cattle with the Muguga cocktail 0801

Animal	Immunization	Days with Schizonts	Days with pyrexia	Days with piroplasms	Day treated	ECF index
BG033	Mug 3308 (M)	5				1.04
BG053	Mug 3308 (M)	2				2.21
BH055	Mug 3308 (M)		2			2.43
BG051	Vac 0801 (C)					1.04
BG042	Vac 0801 (C)					1.04
BH047	Vac 0801 (C)	6	4			2.98
BG056	Vac 0801 (C)					1.04
BG039	None	7	5	2	13	6
BH043	None	7	4	2	13	5.93

All 7 immunized cattle (BG052 succumbed for unknown reasons) and two naïve control cattle BG039, BH043 were challenged with the Muguga cocktail vaccine stabilate 0801. Number of days with schizonts, pyrexia, piroplasms and the days until treatment are indicated. The ECF index is also indicated

## Discussion

It is of major importance to fully understand the mechanisms underlying the protective immune response to *T. parva*, both to underpin the deployment of the current live Muguga cocktail vaccine and to guide the development of a subunit vaccine.

The Muguga cocktail vaccine is composed of parasites from three different isolates (Muguga, Kiambu 5 and Serengeti-transformed), and it is known to protect animals efficiently when deployed in the field [5], despite the presence of antigenic variation in field populations of the parasite [31]. The experiments reported in this article were undertaken to demonstrate that the trivalent Muguga cocktail vaccine provided a more complete protection against heterologous challenge than the Muguga stabilate alone by inducing CTLs capable of recognizing a broader range of parasite strains.

Surprisingly, there was no substantive difference in the CTL responses to the different *T. parva* strains between MHC-matched animals immunized with the Muguga cocktail and the Muguga only. The clonal analysis of one of the haplotype matched pairs supported these results (unfortunately we were not able to generate clones from the other haplotype matched pairs). We were surprised to see the many Kiambu 5-specific clones from the vaccine immunized BG051 animal. This result did not corroborate with the bulk result (Table 2), where there were good responses to Muguga and to Serengeti. It is possible that there is a bias in the cloning process in some cases. Some clones could be easier to expand than others, which would limit the interpretation of the clonal analyses in general. Nevertheless, the bulk results also did not favour a broader CTL specificity in the Muguga cocktail immunized animals compared to those immunized with the Muguga stabilate. An interesting observation was that certain clones recognized many different target cells, which shows that there are indeed broadly cross-reactive antigenic determinants which induce a CTL response following ITM immunization, either with Muguga alone or with the Muguga cocktail. Future work will aim to map these epitopes, which could be useful in a subunit vaccine.

Previous research on the strain specificity of the CTL response in animals immune to *T. parva* suggests that the response in each animal is dominated by a small number of antigens [10, 11, 19, 20]. Clonal analysis of the response in MHC-homozygous animals confirmed this immunodominance by showing that over 60% of the clones from the animals recognized single epitopes in the two respective antigens presented by the MHC haplotypes [24]. The relatively high number of clonotypes observed in the A12/A14 animals analysed here suggests that this is not the case in these animals, unless there is an immunodominant antigen which displays antigenic diversity. In other words, the differential reactivity of the clonotypes is a consequence of the different levels of cross-reactivity of each clone with variant forms of a dominant epitope. The *T. parva* antigens recognized by the various MHC alleles in the A12 and A14 animals have not been fully identified so it is currently not possible to assess the recognition of specific epitopes and the level of diversity displayed by such epitopes. The focus of the response in the Muguga cocktail-immunized animal on Kiambu 5 is interesting and may reflect an immunodominant antigen found predominantly in Kiambu and not shared with many other strains. In this respect, it would be interesting to assess whether CTL from Muguga-only immunized animals are cytotoxic towards the three components in the Muguga cocktail, and to assess whether CTL derived from animals immunized with any one of the components comprising the Muguga cocktail recognize the cloned strains as this may reveal if there are immunodominant, cross-reactive epitopes in the stabilates, and whether this outcome is dependent on the MHC background of the animals.

In order to show if the lack of differences in the CTL responses was reflected in vivo, all animals were challenged with a lethal dose of the Muguga cocktail. There were no major differences in the protection observed in the animals, which indicates that there are no important differences in the antigenic composition of the Muguga stabilate and the Muguga cocktail, at least in animals of the MHC types studied here. This result is perhaps not surprising, as the orginal studies of Radley et al., (1975a) showed that the Muguga stabilate provided good protection against both the Kiambu 5 and Serengeti transformed stabilates, and *vice versa*. In addition, recent deep-sequencing results from our group have also shown that the Muguga cocktail does not contain a great amount of diversity in the known CTL antigens which were examined [32]. Five of the nine antigen genes sequenced were present as a single version, with three present in two forms and the final as three variants. These results suggest that the three components are antigenically very similar and they invoke the question of why the Muguga cocktail provided better protection in the original experiments and why the Muguga cocktail has been so successful in protecting animals against field challenge, where heterogeneous challenges will be far more predominant. It may be, as argued elsewhere [33], that antigenic diversity in the vaccine stabilate is not as essential as originally believed. A note of caution is that most of the antigens which have been sequenced are those presented by predominantly European cattle, and may not reflect important antigens recognized in breeds where the vaccine has been deployed.

These results are somewhat contradictory to the earlier study, where it was shown that a mixture of Muguga, Kiambu5 and Serengeti transformed stabilates provided better protection than individual stabilates [34]. It should be noted that a different challenge strain (Kiambu 1) was used in the earlier experiment. Thus, a possible explanation for the difference in results is that the CTL induced by the Muguga isolate do not cross-react with antigens present in Kiambu 1, at least in the animals of the MHC types used in the earlier experiment. This experiment was performed before the role of CTL as the mediators of immunity and the influence of the MHC were established, and the MHC types of the animals are not available.

However, the difference in the results does indicate that, although we have shown that Muguga-immunized animals of the MHC types used here can generate CTL of similar strain specificities as those immunized with the Muguga cocktail, we cannot with certainty state that use of the Muguga isolate alone will provide the same broad protection in the field as the Muguga cocktail appears to provide. In this respect, it would also be interesting to test if cattle immunized with either Muguga only or the Muguga cocktail would be protected if challenged with the strains, that previously were shown to break through single-strain immunization, such as Marikebuni and Kiambu 1, this time with a larger number of cattle per group.

The field situation is far more complicated than the experimental conditions employed here, due to the presence of a much more heterogenous population of parasites and the diversity of MHC types in outbred cattle populations of several breeds. Both of these factors threaten the success of vaccines composed of a limited number of parasite strains. Particularly at risk are cattle populations in the buffalo-cattle interface, as parasites derived from buffalo show much greater antigenic diversity than those from cattle [31]. Indeed, it has been shown that immunization with the Muguga cocktail does not protect cattle in areas of close interaction with buffalo [35], although it has not been established that this is due to antigenic diversity. Close monitoring of break-through incidences in the field with use of the Muguga cocktail versus single strains would show whether or not single strains protect as well as the Mugaga cocktail.

## Conclusion

There were no indications that a broader immune response was induced by immunization with the Muguga cocktail compared to the Muguga strain only, in the haplotypes examined in this study. In agreement with this, previous studies on antigenic diversity in the Muguga cocktail found limited diversity. As the original studies using single strain and Muguga cocktail for induction of protection were performed with limited number of animals and with high doses of needle challenges, this may warrant for testing single vaccine strains in field settings where the load of parasites during challenge will be much lower.

## Abbreviations

ConA: Concanavalin A
CTL: Cytotoxic T Lymphocytes
FBS: Fetal Bovine Serum
IFNγ: Interferon Gamma
ITM: Infection and Treatment Method
mAb: Monoclonal Antibody
MHC: Major Histocompatibility Complex
PBMC: Peripheral Blood Mononuclear Cells
SD: Standard Deviation
TCGF: T-Cell Growth Factor
TCR: T Cell Receptor
